# Effects of Ultrasonic Treatment on the Microstructure and Mechanical Properties of Mg-3Y-3.5Sm-2Zn-0.6Zr (wt %) Alloy

**DOI:** 10.3390/ma12172722

**Published:** 2019-08-25

**Authors:** Minghua Liu, Miaomiao Li, Along Wang, Mengqi Zhang, Wei Wang, Wenli Wang

**Affiliations:** School of Metallurgical Engineering, Xi’an University of Architecture and Technology, Xi’an 710055, China

**Keywords:** magnesium, ultrasonic treatment, microstructure, mechanical properties

## Abstract

Ultrasonic treatment (UST) was applied in the smelting process of Mg-3Y-3.5Sm-2Zn-0.6Zr (wt %) alloy and the microstructure as well as mechanical properties of the experimental alloy were investigated. Results showed that the effect of UST on grain refinement was obvious, and the distribution of the second phases along grain boundary became discontinuous. The width of the grain boundary precipitates decreased after UST. The contents of solute elements within grains increased, and the morphologies of Zr-rich compounds and Y-rich compounds both at grain boundaries and within grains changed after UST. The mechanical properties of the experimental alloy after UST were significantly improved. The ultimate tensile strength (UTS) was 265 MPa, the tensile yield strength (TYS) was 171 MPa, and the elongation (EL) was 11%. The mechanism of UST of the alloy can be attributed to the combined effects of cavitation-induced heterogeneous nucleation and melt convection induced by acoustic streaming, resulting in the refinement of grains and the grain boundary precipitates, which promoted the improvement of mechanical properties.

## 1. Introduction

Magnesium alloys have attracted significant attention in the fields such as aviation, aerospace, transportations, chemicals, 3C (computer, communication, and consumer electronics), and other industries owing to their low density, high specific strength and stiffness, good damping capacity, excellent machinability, and easy recovery [1]. However, the strength, ductility, and heat resistance at high temperatures of magnesium alloys are still not good enough for a more broad-range of commercial applications [2]. Grain refinement is an important way to improve the comprehensive mechanical properties of magnesium alloys [3]. It can be carried out through two different methods, including chemical method and physical method. As grain refiners, chemical elements (C, Zr, and Ca) are added into magnesium alloy to refine the grain size [4]. According to literature [5], the grain size can be refined from 60 to 19.6 mm by adding element Zr into as-cast Mg–Zn–Zr–Sr alloy. According to literature report [6], mechanical properties of the Mg–Zn–Ca alloy are significantly improved after the addition of Zr. As a simple, clean, and effective physical method, ultrasonic treatment (UST) has considerable potential for refining the grain size and modifying the microstructure of magnesium alloys during solidification process [7,8,9]. UST has been widely introduced into light alloys, such as aluminum alloys and magnesium alloys. The nonlinear effects such as cavitation and sound streaming induced by the introduction of UST in the alloy melt promote melt agitation and homogenization, thereby increasing the convection diffusion rate and improving the microstructure of the alloy [10]. Previous investigations have clearly demonstrated that UST can be efficiently introduced into the melts and shows promising effects on grain refinement and mechanical properties of the solidified alloys [11,12,13]. Yang et al. [14] significantly refined and homogenized the LPSO structure in Mg_99.0 − x_Ni_x_Y_1.0_ (x = 0.5, 1.0, 1.5, at %) alloys by UST. Moussa et al. [15] found that UST could reduce the grain size and modify the primary Mg_2_Si during the solidification process. Zhang et al. [16] found that UST could break the dendritic phases into pieces and improve the mechanical properties of A356 alloy. According to literature [17], with the application of UST, the number of particles actually responsible for nucleating grains and the potency of nuclei increased and the average grain size of all the AZ alloys decreased by about 70–80%. 

As mentioned above, the effects of chemical element Zr and UST on the microstructure and mechanical properties of magnesium alloys have been investigated separately. However, the effect of UST on the microstructure and mechanical properties of Zr-containing magnesium alloys has rarely been investigated. In this study, UST was applied in the smelting process of Mg-3Y-3.5Sm-2Zn-0.6Zr (wt %) alloy and the microstructure as well as mechanical properties of the experimental alloy were investigated. Finally, the mechanism of UST was comprehensively discussed.

## 2. Materials and Methods 

**Figure 1:**
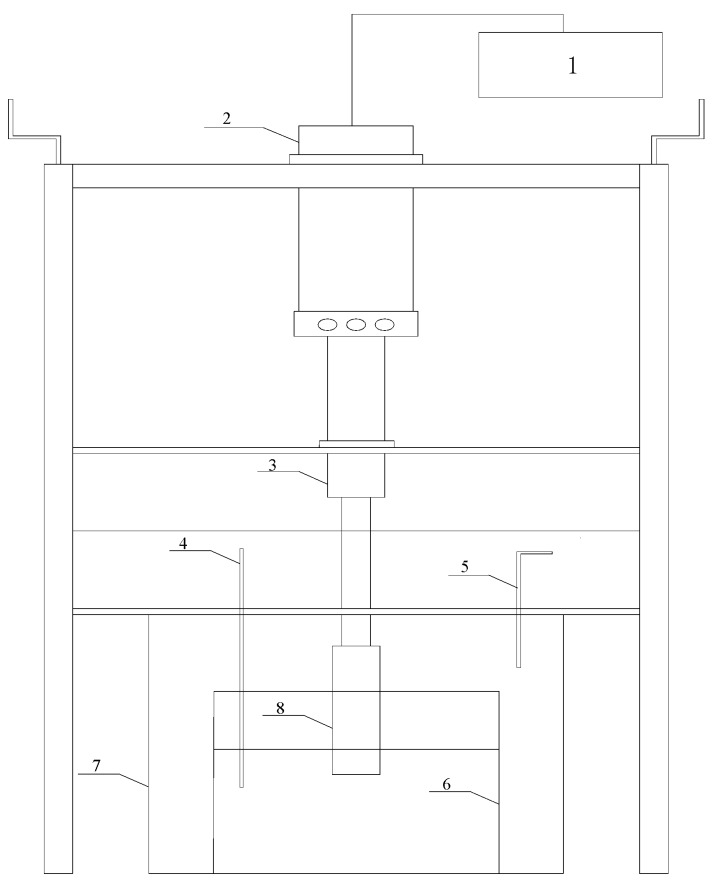
Schematic illustration of the experimental set-up: **1** Ultrasonic generator, **2** Transducer, **3** Horn, **4** SF_6_+CO_2_, **5** Thermocouple, **6** Stainless steel crucible, **7** Resistance furnace, **8** Tool head.

Figure 1 shows the schematic illustration of experimental device which consists of TJS-Intelligent generator V2.0 (Hangzhou Success Ultrasonic Equipment Co., Hangzhou, China) with a fixed frequency of 20 kHz, an ultrasonic probe made of titanium alloy, a resistance furnace, and a thermocouple. The ultrasonic generator power is continuously adjustable from 0 to 2000 W, and the output was automatically adjusted according to the load size.

Mg-3Y-3.5Sm-2Zn-0.6Zr alloy was used in this study. The raw materials included pure Mg (99.99 wt %) ingot, Mg–Zr master alloy (with 30 wt % Zr), Mg–Y master alloy (with 30 wt % Y), Mg–Sm master alloy (with 20 wt % Sm) and pure Zn (99.99 wt %) ingot. The melting process of the experimental alloy was carried out in an electrical resistance furnace. In order to avoid melt burning, 1% (volume fraction) SF_6_ + 99% (volume fraction) CO_2_ atmosphere was introduced to protect the melt. The total weight of the alloy was 1 kg. The preheated Mg was added into the crucible at 750 °C and then the pure Zn ingot, Mg–Y master alloy, and Mg–Sm master alloy were subsequently added into the melt. The Mg–Zr master alloy was added into the melt when the temperature increased to 780 °C, and then the melt were held at 780 °C for 25 min. When the melt temperature reduced to 700 °C, the ultrasonic horn was immersed into the melt and carried out UST for 90 s. Then the ultrasonic probe was quickly removed. The melt was cast into a preheated steel mold and cooled with water. The nominal chemical composition of the as-cast Mg-3Y-3.5Sm-2Zn-0.6Zr alloy (wt %) is presented in Table 1.

**Figure 2:**
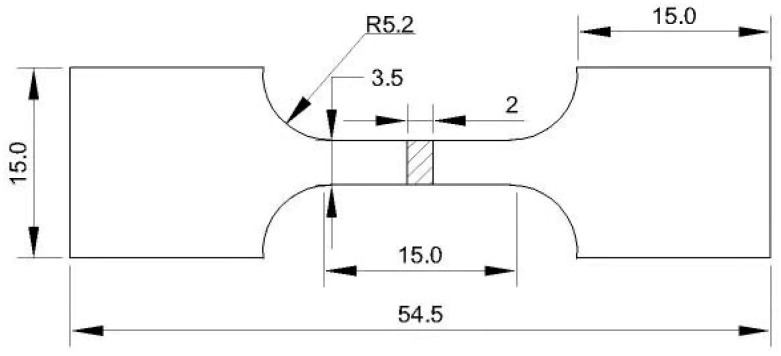
Drawing of tensile sample (unit: mm).

The as-cast samples were cut, sanded, mechanically polished and etched in a solution of 3–5 g picric, 5 mL glacial acetic acid, 10 mL distilled water and 90 mL ethanol to reveal their microstructures. Microstructure characterizations of the specimens were identified by the OLYMPUS GX51 type metallographic microscope (Olympus, Tokyo, Japan) and the JEOL JSM-6390A type scanning electron microscope equipped with energy dispersive X-ray spectroscopy (EDS, Karl Zeiss, Jena, Germany). The size of the specimens used for microstructural characterizations observation was 10 mm × 10mm × 15 mm. The average grain size was calculated by following the mean linear intercept method in ImageJ software whose version number is 1.48. Phase analysis was carried out by the BRUKER D8 ADVANCE type X-ray diffractometer (XRD, Bruker AXS company, Karlsruhe, Germany). The mechanical properties at room temperature were tested at a constant strain rate of 0.5 mm min^−1^ on the INSTRON8801 type servohydraulic testing machine (INSTRON Corporation, Norwood, MA, USA). The maximum load of this machine was 50 KN and its working frequency was less than 50 Hz. Figure 2 shows the drawing of tensile sample, where the total length and thickness of the tensile sample were 54.5 mm and 2 mm, respectively. Differential scanning calorimetry (DSC) curve was obtained by TGA/DSC3+ type synchronized thermal analyzer (METTLER, Urdorf, Switzerland), and the cooling rate was 10 °C min^−1^. DSC specimen with size of 2 mm × 2 mm × 2 mm was processed from the casting, and the experiment was carried out in a protective atmosphere of high purity nitrogen. 

## 3. Results

### 3.1. Microstructures

**Figure 3:**
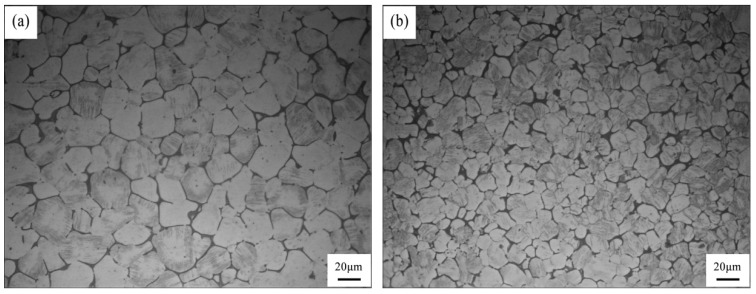
Optical microstructures of the as-cast Mg-3Y-3.5Sm-2Zn-0.6Zr alloy: (**a**) Without UST; (**b**) with UST.

Figure 3 shows the representative microstructures of the as-cast Mg-3Y-3.5Sm-2Zn-0.6Zr alloy with and without UST. The alloy without UST consisted of equiaxed grains, second phase precipitates at grain boundaries, some spherical particles, and the average grain size was about 20 μm, as shown in Figure 3a. The grains of the alloy treated by UST were refined and the average size decreased to about 14 μm, as shown in Figure 3b.

**Figure 4:**
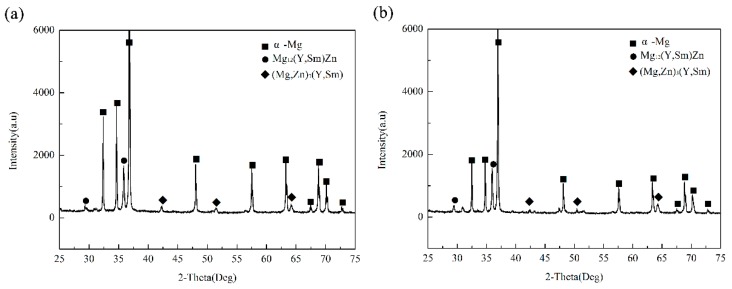
XRD patterns of the as-cast Mg-3Y-3.5Sm-2Zn-0.6Zr alloy: (**a**) Without UST; (**b**) with UST.

**Figure 5:**
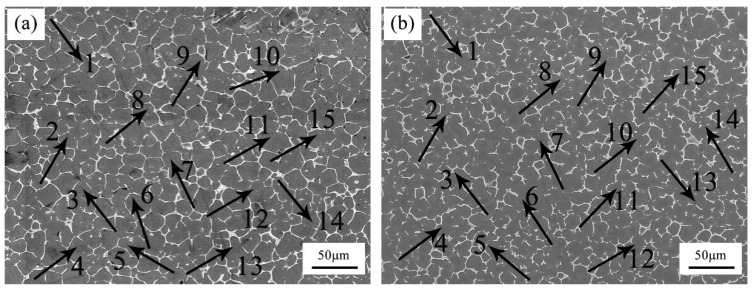
SEM and EDS data acquisition images of the as-cast Mg-3Y-3.5Sm-2Zn-0.6Zr alloy: (**a**) Without UST; (**b**) with UST.

Figure 4 shows the XRD patterns of the as-cast Mg-3Y-3.5Sm-2Zn-0.6Zr alloy under different conditions. The results showed that the alloys consisted of α-Mg, Mg_12_(Y,Sm)Zn and (Mg,Zn)_3_(Y,Sm), indicating that UST did not change the phase composition of the alloy. Figure 5 shows the SEM images of the as-cast Mg-3Y-3.5Sm-2Zn-0.6Zr alloy without and with UST. Microstructure of the alloy without UST consisted of coarse and semicontinuous net phases which distribute mainly at the grain boundaries, as shown in Figure 5a. However, after UST, the continuity of the grain boundaries precipitates decreased, and the number of rod-like precipitates with length of 5–10 μm increased, as shown in Figure 5b. Therefore, the morphology of grain boundaries precipitates changed and the number of coarse precipitates decreased after UST. 

**Figure 6:**
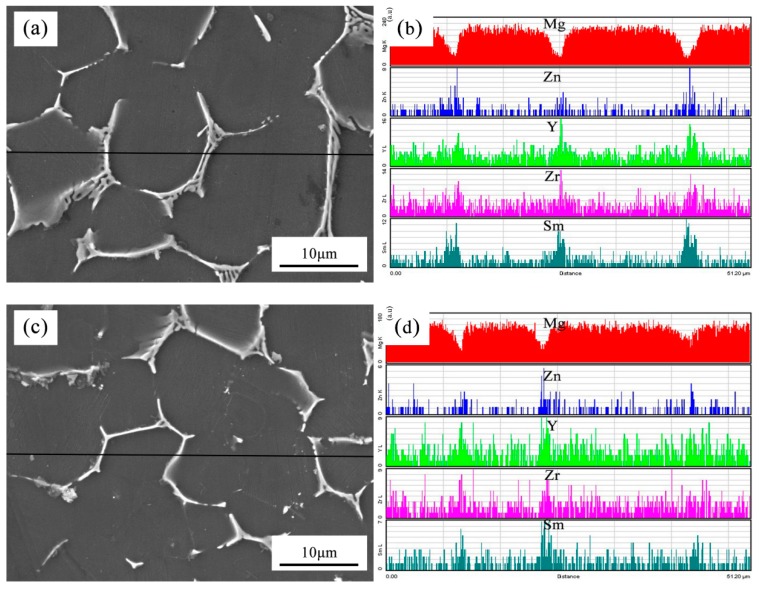
Line scanning images of the as-cast Mg-3Y-3.5Sm-2Zn-0.6Zr alloy: (**a**,**b**) Without UST; (**c**,**d**) with UST.

**Figure 7:**
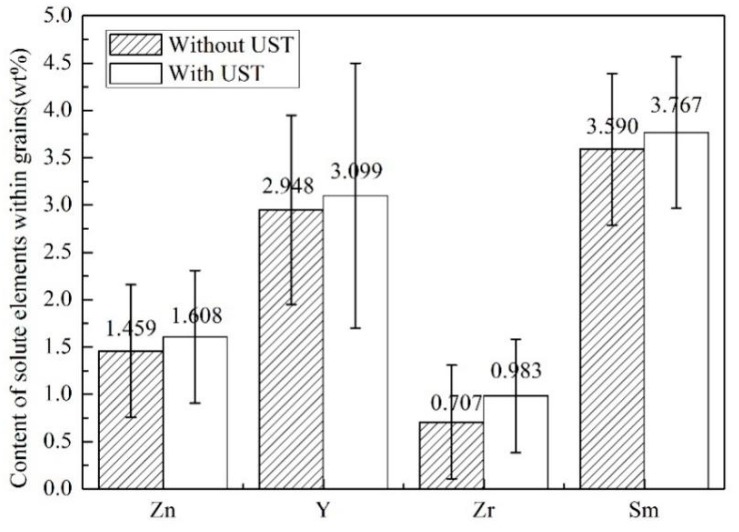
**** Content of solute elements within grains.

Figure 6 shows the line scanning results of the as-cast Mg-3Y-3.5Sm-2Zn-0.6Zr alloy under different conditions. The transverse axis of Figure 6b,d was the line scanning length selected in the experiment, and its unit was μm. The linear scanning length of both untreated and treated alloys was 5120 μm. Vertical axis represented the characteristic X-ray counting intensity of elements, and its unit was a.u. Similar to Figure 4, a number of precipitates existed at the grain boundaries of untreated and treated alloys, which indicated that the solute elements in the alloys segregated at the grain boundaries, as shown in Figure 6. However, as can be seen from Figure 6a,c, the width size of the grain boundary precipitates decreased after UST, which was also reflected by the differences in the vertical axis values of Figure 6b,d. In order to study the distribution of solute elements in the alloy, 15 points within grains were randomly selected and analyzed by EDS. Table 2 lists the average contents of these solute elements within grains. Figure 7 exhibits the content of solute elements within grains of the 15 points shown in Figure 5. The contents of Zr and Sm were slightly higher than those of nominal components under the two conditions of without UST and with UST. Moreover, the content of element Y under UST was slightly higher than that of nominal component. These phenomena were due to the difference between the burning loss rate used in preparing raw materials and the actual burning loss rate. In addition, the contents of solute elements Zn, Y, Zr, and Sm within grains increased after UST, as shown in Figure 7. Obviously, UST reduced the width size of the grain boundary precipitates (Figure 6) and promoted the dissolution of solute elements in the matrix (Figure 7). 

**Figure 8:**
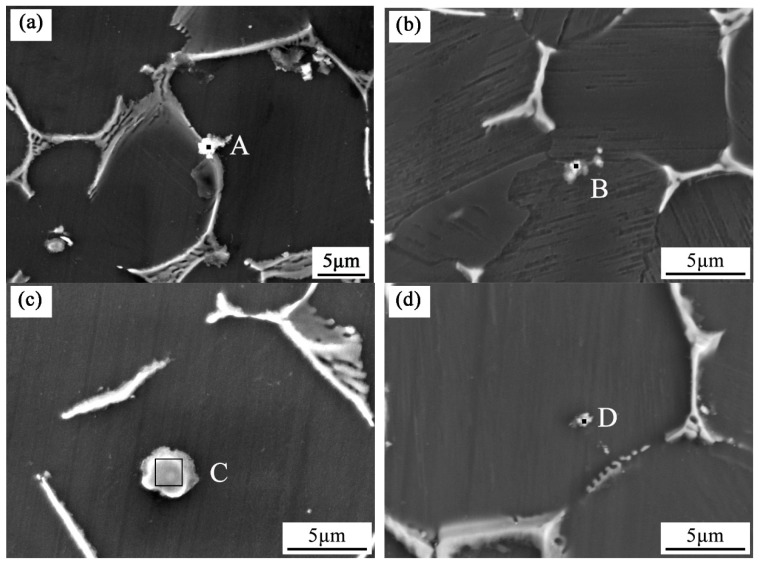
**** High magnification SEM images of Zr in the as-cast Mg-3Y-3.5Sm-2Zn-0.6Zr alloy: (**a**,**c**) Without UST; (**b**,**d**) with UST.

**Figure 9:**
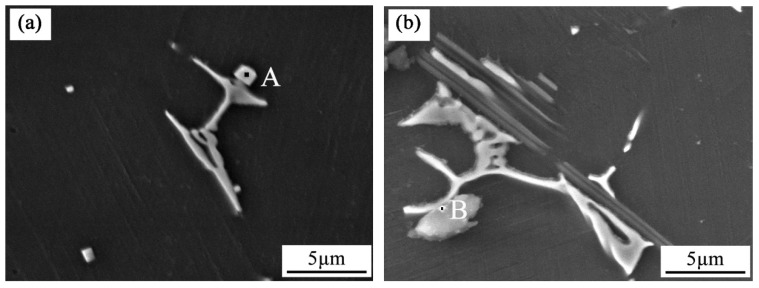
**** High magnification SEM images of Y-rich compound in the as-cast Mg-3Y-3.5Sm-2Zn-0.6Zr alloy: (**a**) Without UST; (**b**) with UST.

Figure 8 shows the high magnification SEM images of Zr in the as-cast Mg-3Y-3.5Sm-2Zn-0.6Zr alloy without and with UST. Table 3 and Table 4 show EDS results corresponding to the selected points in Figure 8. Combining Figure 8 and Table 3 and Table 4, it can be seen that some Zr-rich compounds were observed both at grain boundaries and within grains. After UST, Zr-rich compounds became smaller in size and more dispersed in distribution. As shown in Figure 8a, the Zr-rich compound at the grain boundary agglomerated into bright white spherical particles with a size of approximately 2.5 μm. With the application of UST in the melt, the Zr-rich compound turned into a grayish, scattered block having a size of about 1.7 μm, as shown in Figure 8b. Figure 8c,d show a comparison of the microstructures of Zr-rich compounds distributed within grains. Figure 8c exhibits the morphology of the Zr-rich compound. The Zr-rich compound consisted of a gray circular halo and a white ring, which was different from the morphology observed in Figure 8a. The size of the Zr-rich compound was about 3.0 μm. Moreover, the Zr-rich compound shown in Figure 8d was much smaller than that shown in Figure 8c. Figure 9 and Table 5 and Table 6 show the high magnification SEM images and EDS results of Y-rich compound. The size of Y-rich compound shown in Figure 9a was similar to the gray–white cubic block with side lengths of 0.7–1.0 μm. After UST, as shown in Figure 9b, the Y-rich compound became a small dot with diameter of about 0.4 μm. Clearly, UST introduced into the melt changed the morphologies of Zr-rich compounds and Y-rich compounds (Figure 8 and Figure 9).

### 3.2. Mechanical Properties and Fracture Analysis 

**Figure 10:**
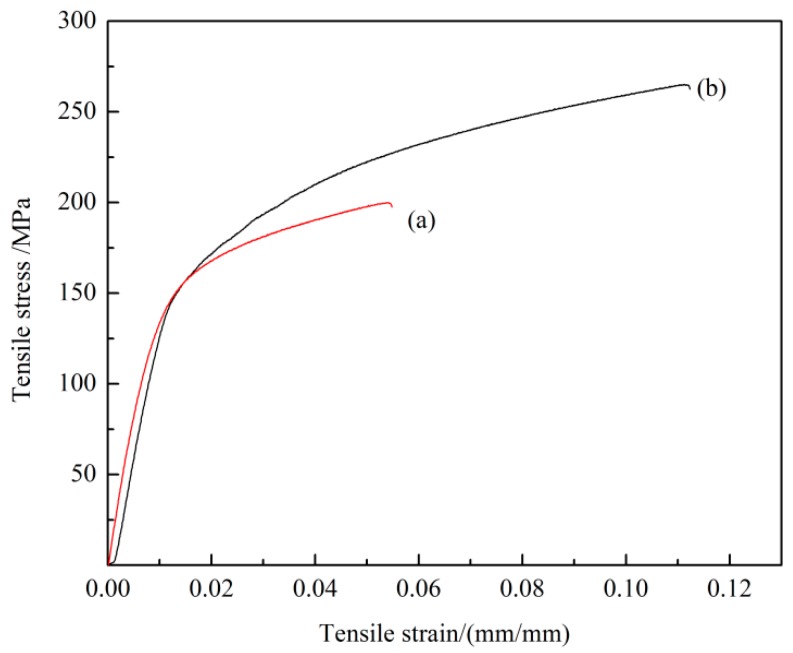
**** Room-temperature tensile stress-strain curves of the as-cast Mg-3Y-3.5Sm-2Zn-0.6Zr alloy and: (**a**) Without UST; (**b**) with UST.

Room-temperature tensile stress-strain curves of the as-cast Mg-3Y-3.5Sm-2Zn-0.6Zr alloy without and with UST are shown in Figure 10. Obviously, UST significantly influenced the mechanical properties of Mg-3Y-3.5Sm-2Zn-0.6Zr alloy. Without UST, the ultimate tensile strength (UTS), tensile yield strength (TYS), and elongation (EL) of the as-cast Mg-3Y-3.5Sm-2Zn-0.6Zr alloy were 200 MPa, 160 MPa, and 5%, respectively. With the application of UST, the mechanical properties of the Mg-3Y-3.5Sm-2Zn-0.6Zr alloy were significantly improved. The values of the UTS, TYS, and EL increased to 265 MPa, 171 MPa, and 11%, respectively.

**Figure 11:**
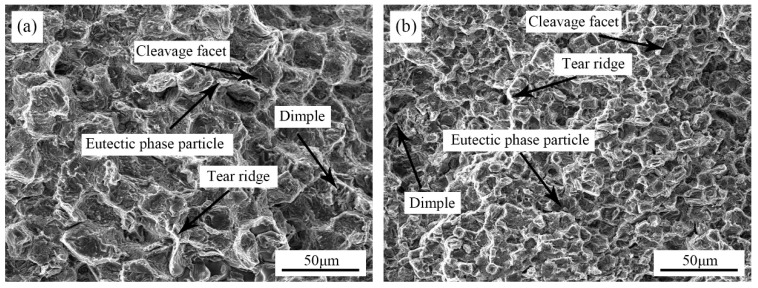
Fractures of the as-cast Mg-3Y-3.5Sm-2Zn-0.6Zr alloy: (**a**) Without UST; (**b**) with UST.

Figure 11 shows the SEM images of fracture surfaces of the as-cast Mg-3Y-3.5Sm-2Zn-0.6Zr alloy. The fracture surfaces of the alloys were composed of cleavage facets, tear ridges, eutectic phase particles and dimples. Grains on the fracture were ice-sugar-like structures. Eutectic phase particles at grain boundaries became the source of cracks, and the propagation of cracks along grain boundaries led to intergranular fracture. Therefore, the fracture mode of the alloy without UST was mainly intergranular fracture. For the alloy treated by UST, the fracture mode was still intergranular fracture. However, the area of cleavage facets decreased after UST, and the number of tear ridges around cleavage facets increased. The amount of dimples also increased after the UST. Furthermore, with the application of UST, the shape of the dimples changed from shallow groove shape to deeper cup shape. Moreover, the second phase particles on the fracture surface became smaller after UST, which reduced the generation of crack sources and was beneficial to the improvement of mechanical properties of the alloy. Thus the alloy with UST exhibited a relatively high elongation.

## 4. Mechanism Discussion

Applying UST to the melt generates cavitation and acoustic streaming effect [18,19,20]. When the ultrasonic wave propagates in the melt, the liquid molecules in the sparse region of the sound wave are subjected to tensile stress under the action of the periodic alternating sound field, which then crack to form cavitation bubbles. The cavitation bubbles undergo a process of formation, expansion, and collapse, and the resulting cavitation effect can effectively refine the alloy solidification structure. Acoustic streaming effect produced during the introduction of UST can enhance the convection of alloy melt and promote the uniform distribution of composition of the melt and grain refinement as well. 

Dendrite fragmentation caused by cavitation is more effective and dominant when the temperature of UST is lower than the liquidus temperature [21,22]. This is attributed to the fact that the solid fraction of the alloy increases as the melt begins to solidify, and the shear stress produced by the collapse of cavitation bubbles can break the dendrites. These broken dendrites redistribute to the melt under the action of acoustic streaming, thus increasing the number of nucleations in the melt. The relationship between the fracture length l and radius r of dendrite and the required pressure is P_d_ = 0.25(r/l)^2^ σ_mp_, where σ_mp_ is the shear strength of materials close to the melting point [21]. 

**Figure 12:**
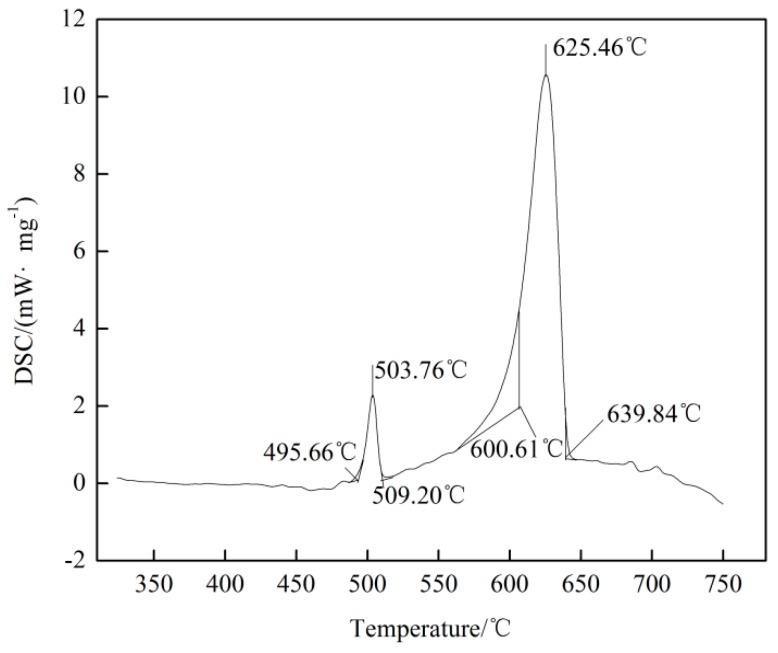
**** DSC cooling curve of the as-cast Mg-3Y-3.5Sm-2Zn-0.6Zr alloy.

Figure 12 exhibits DSC cooling curve of the as-cast Mg-3Y-3.5Sm-2Zn-0.6Zr alloy. During DSC testing process, the temperature of the experimental alloy decreased from 750 °C to 300 °C at a rate of 10 °C min^−1^. When the temperature dropped to 639.84 °C, formation of the α-Mg solid solution initiated exothermically. At 625.46 °C, the heat release reached its peak value. Eutectic reaction occurred with a lot of heat released at 509.20 °C. The temperature of exothermic peak was 503.76 °C. Moreover, the liquidus temperature of the alloy was 639.84 °C, as shown in Figure 12.

In this study, the UST temperature was 700 °C, which was higher than the liquidus temperature of the alloy. Therefore, dendrite fragmentation is not the main factor for grain refinement of microstructure in this study.

Grain refinement caused by UST performed above the liquidus temperature is mainly related to heterogeneous nucleation enhanced by cavitation, which can be explained in terms of three different mechanisms [9,17,23]. First mechanism proposes that the melt inside the cavitation bubbles evaporates and absorbs heat from the surrounding during the expansion of cavitation bubbles, which reduces the surface temperature of the bubbles and forms local undercooling, leading to the formation of nuclei on the surface of the bubbles [9,10,24]. When the cavitation bubbles collapse, these nuclei are distributed into the melt under the action of acoustic streaming. The interaction of cavitation and acoustic streaming leads to the increase in the nucleation rate and leads to the decrease of the grain size. In the present work, the average grain size of the as-cast Mg-3Y-3.5Sm-2Zn-0.6Zr alloy decreased after UST (Figure 3), which can be explained by the first mechanism. In addition, the melt convection caused by acoustic streaming changed the distribution of solute elements in the alloy (Figure 7). Second mechanism [20,25] assumes the presence of a large number of insoluble non-metallic inclusions (intermetallic compounds, oxides, etc.) in the melt. Usually these inclusions cannot be wetted by the melt. The high-pressure pulse generated by cavitation collapse can effectively fill the melt into the surface defects and microcracks of inclusions. Therefore, the wettability of these inclusions is improved, making them effective nucleating substrates. It is noteworthy that Zr-rich compounds and Y-rich compounds were observed in this study. The application of UST in the alloy enhanced the wettability of these compounds. Furthermore, the collapse of the cavitation bubbles and the melt convection caused by the acoustic streaming resulted in decrease in their size, and the morphology changed from agglomeration to dispersion (Figure 8 and Figure 9). The third mechanism [21] is pressure pulse melting point (Tm) mechanism. According to the Clausius–Clapeyron equation, the pressure pulse induced by the collapse of cavitation bubbles increases Tm [26]. Clausius–Clapeyron equation: dTm /dP=Tm (V_L_ − V_S_)/ΔH, where Tm represents the freezing point, P refers to pressure, V_L_ and V_S_ are the specific volume of liquid and solid phase with the unit of cm^3^ g^−1^, respectively. ΔH is the latent heat of freezing. The increase of Tm is equivalent to the increase of undercooling, which is beneficial to the improvement of nucleation rate. The third mechanism is more suitable for pure metals and is not suitable for magnesium alloys with a wide melting temperature range [26]. Therefore, it is possible that the first two mechanisms act alone or in combination to refine grains of the as-cast Mg-3Y-3.5Sm-2Zn-0.6Zr alloy.

Moreover, Figure 10 exhibits the improvement in the mechanical properties of the as-cast Mg-3Y-3.5Sm-2Zn-0.6Zr alloy after UST. The following two reasons can be used to explain this phenomenon. First, UST led to the remarkable grain refinement effect in the experimental alloy. The tensile strength increased with the decrease of grain size [3]. Moreover, the refinement of the as-cast Mg-3Y-3.5Sm-2Zn-0.6Zr alloy inhibited twin deformation and increased the amount of slip deformation [27], which can be beneficial to improving the plasticity of the alloy. Second, the continuity of the grain boundaries precipitates reduced (shown in Figure 5) and the width size of the grain boundary precipitates decreased (shown in Figure 6) after UST. These two factors hindered the occurrence of crack in tensile test and promoted the improvement of mechanical properties of alloys. As a result, the mechanical properties of the as-cast Mg-3Y-3.5Sm-2Zn-0.6Zr alloy produced with UST were improved.

## 5. Conclusions

In this study, the effects of the application of UST on the microstructure and mechanical properties of Mg-3Y-3.5Sm-2Zn-0.6Zr alloy were investigated by using relevant experimental equipment. The main conclusions are as follows.

After UST, the average grain size of the as-cast Mg-3Y-3.5Sm-2Zn-0.6Zr alloy decreased from 20 to 14 μm. The continuity of the grain boundary precipitates reduced and the width size of them decreased after UST. The contents of solute elements within grains increased, and the morphologies of Zr-rich compounds and Y-rich compounds both at grain boundaries and within grains changed after UST.

Without UST, the values of UTS, TYS, and EL of the as-cast Mg-3Y-3.5Sm-2Zn-0.6Zr alloy were 200 MPa, 160 MPa, and 5%, respectively. With the application of UST, the mechanical properties of the alloy were significantly improved. The values of the UTS, TYS and EL increased to 265 MPa, 171 MPa, and 11%, respectively.

The mechanism of UST in the alloy can be attributed to the single or combined effects of cavitation-induced heterogeneous nucleation and melt convection induced by acoustic streaming, which resulted in the refinement of grains and the grain boundary precipitates. Therefore, the mechanical properties of the alloy were improved.

## Figures and Tables

**Table 1:** Nominal chemical composition of the as-cast Mg-3Y-3.5Sm-2Zn-0.6Zr alloy (wt %).

Alloy	Zn	Y	Zr	Sm	Mg
Mg-3Y-3.5Sm-2Zn-0.6Zr	2.0	3.0	0.6	3.5	Bal.

**Table 2:** The average contents of main solute elements within grains (wt %).

Alloy	Zn	SD	Y	SD	Zr	SD	Sm	SD	Mg
Without UST	1.459	0.7	2.948	1.0	0.707	0.6	3.590	0.8	Bal.
With UST	1.608	0.7	3.099	1.4	0.983	0.6	3.767	0.8	Bal.

**Table 3:** EDS results corresponding to the selected points in Figure 8 (wt %).

Points	Zn	Y	Zr	Sm	Mg
**A**	3.13	4.12	61.59	5.98	Bal.
**B**	1.62	3.56	66.05	2.49	Bal.
**C**	1.23	2.79	94.53	0.77	Bal.
D	3.48	3.58	76.93	1.79	Bal.

**Table 4:** EDS results corresponding to the selected points in Figure 8 (at %).

Points	Zn	Y	Zr	Sm	Mg
**A**	2.60	2.51	36.59	2.15	Bal.
**B**	1.31	2.12	38.38	0.88	Bal.
**C**	1.69	2.80	92.56	0.46	Bal.
D	3.47	2.63	55.00	0.78	Bal.

**Table 5:** EDS results corresponding to the selected points in Figure 9 (wt %).

Points	Zn	Y	Zr	Sm	Mg
**A**	0.68	61.08	6.07	21.41	Bal.
B	6.00	33.14	2.14	11.59	Bal.

**Table 6:** EDS results corresponding to the selected points in Figure 9 (at %).

Points	Zn	Y	Zr	Sm	Mg
A	0.77	50.92	4.93	10.55	Bal.
B	3.66	14.88	0.94	3.08	Bal.
